# Physiological and muscle tissue responses in *Litopenaeus vannamei* under hypoxic stress *via* iTRAQ

**DOI:** 10.3389/fphys.2022.979472

**Published:** 2022-08-30

**Authors:** Fengtong Chang, Na Li, Xiang Shi, Volovych Olga, Xiaobing Wang, Xiaoping Diao, Hailong Zhou, Xianming Tang

**Affiliations:** ^1^ State Key Laboratory of South China Sea Marine Resource Utilisation, Hainan University, Haikou, China; ^2^ School of Life Sciences, Hainan University, Haikou, China; ^3^ Hainan Provincial Key Laboratory of Tropical Maricultural Technology, Hainan Academy of Ocean and Fisheries Sciences, Haikou, Hainan, China; ^4^ One Health Institute, Hainan University, Haikou, Hainan, China

**Keywords:** *Litopenaeus vannamei*, hypoxia stress, physiological responses, proteome, real-time quantitative PCR

## Abstract

White *L. vannamei* have become the most widely cultivated shrimp species worldwide. Cultivation of *L. vannamei* is one of the predominant sectors in China’s aquaculture industry. This study focused on the physiological and biochemical responses, differential protein expression, and expression characteristics of the related crucial functional protein genes under low oxygen conditions among different strains of *L. vannamei*. It was found that 6 h of hypoxic stress caused a significant reduction in the total hemocyte number in both strains, while the hypoxia-sensitive strain showed a stronger reduction. In contrast, the hemocyanin concentration showed only an overall upward trend. Proteomic analysis of *L. vannamei* muscle tissue revealed 3,417 differential proteins after 12 h of hypoxic stress. Among them, 29 differentially expressed proteins were downregulated and 244 were upregulated in the hypoxia-sensitive strain. In contrast, there were only 10 differentially expressed proteins with a downregulation pattern and 25 with an upregulation pattern in the hypoxia-tolerant strain. Five protein genes that responded significantly to hypoxic stress were selected for quantitative real-time PCR analysis, namely, hemocyanin, chitinase, heat shock protein 90 (HSP 90), programmed death protein, and glycogen phosphorylase. The results showed that the gene expression patterns were consistent with proteomic experimental data except for death protein and glycogen phosphorylase. These results can enrich the general knowledge of hypoxic stress in *L. vannamei* and the information provided differentially expressed proteins which may be used to assist breeding programs of *L. vannamei* of new strains with tolerance to hypoxia.

## Introduction

Dissolved oxygen (DO) is an important factor in the aquatic environment and an indicator of water quality. The concentration of DO in water is normally approximately 6 mg/L, while lower than 2.8 mg/L is referred hypoxia (Diaz and Rosenberg 1995). Global climate change and human activities intensified hypoxic conditions in marine ecosystems especially in coastal areas around the world (Breitburg et al., 2018). The expansion of hypoxic areas in the open ocean and coastal waters are expected to continue and will have a great impact on the ecosystem and biodiversity (Keeling, Kortzinger and Gruber 2010).

Animal responses to hypoxic stress are divided into physiological, biochemical, and behavioral responses. A physiological response includes changes in heart rate, respiratory metabolism, cell proliferation, and apoptosis, hemocyanin level, immune response, antioxidant capacity, and osmotic regulation ability. Hypoxic environments may also alter the expression of certain genes that subsequently leads to a series of biochemical and physiological responses, which allow organisms to survive in such conditions.

It's been reported that hypoxia can elicit adverse effects on the behavior, growth, development, respiration, metabolism, immunity, DNA damage, and gene expression of aquatic organisms (Levin et al., 2009; Guadagnoli, Tobita and Reiber 2011; Zhou et al., 2014; Li et al., 2016). Various studies found that the rate of growth, weight gain, and feed utilization in channel catfish (*Ictalurus punctatus*), Atlantic cod (*Gadus morhua*), fish (*Leporinus elongates*), and silver catfish (*Rhamdia quelen*) were decreased under hypoxic conditions (Buentello et al., 2000; Chabot and Biology. 2005; Filho et al., 2005; Riffel et al., 2014). An extensive study of red-eared turtles under hypoxic stress revealed changes in carbohydrate metabolism and the antioxidant defense system. Along with the increase in hypoxic duration, the contents of lactic acid and blood glucose in the blood increased rapidly, while the glycogen in skeletal muscle and liver was gradually consumed (Ye et al., 2011).

According to a report by Gray, Wu and Ying (2002), the anoxic resistance of aquatic animals decreases in the following order: mollusks, annelids, echinoderms, crustaceans, and fish. Shrimp and other marine invertebrates lack adaptive immune mechanisms and rely on innate immune responses to cope with environmental stress (Tassanakajon et al., 2018). Currently, research on the effects of hypoxia on prawns and other crustaceans is mainly focused on changes in innate immune system parameters, such as total hemocyte counts (THCs) and hemocyanin concentrations (HCs), which could reveal possible adaptations to hypoxic conditions. Chen *et al.* found that the THC of scallop (*Chlamys farreri*) gradually and significantly decreased with a decline in the DO level to 2.5 mg/L (Chen et al., 2007). A similar study was performed on freshwater prawn (*Macrobrachium rosenbergii*) and revealed a reduction in THC by 36% after 12 h in a 2.75 mg/L DO concentration environment (Cheng and Chen 2000). In response to hypoxia, shrimp can increase their HC to maintain oxygen transport. In a related study, two strains of *L. vannamei* had significantly decreased THCs under hypoxic stress, while having significantly increased HCs (Wei Y et al., 2016).

The rapid development of proteomics technology in recent years has allowed its wide application in the study of aquatic crustacean pathogen infection (Chongsatja et al., 2007; Wang et al., 2007). For example, a combination of two-dimensional (2-DE) electrophoresis, mass spectrometry, and bioinformatics tools was used to discover the major allergen of freshwater prawn (*Macrobrachium rosenbergii*) (Yadzir et al., 2012). However, only a few studies have reported the hypoxic stress response in crustaceans using this approach, one of which is the investigation of hypoxia effects on oriental river prawn (*Macrobrachium nipponense*) muscle proteome using a 2D-gel-based proteomics approach coupled with mass spectrometry (MS) (Sun et al., 2016).

In marine crustaceans, changes in gene expression often underlie or reflect key physiological and biochemical acclimations to hypoxia, which has been verified by global transcriptome profiling by microarrays (Li and Brouwer, 2013). Crustaceans also respond to hypoxia by altering levels of respiratory pigments, antioxidant proteins, and enzymes involved in glycolysis, amino acid and nucleotide metabolism (Brouwer et al., 2004; Abe et al., 2007; Jiang et al., 2009). Particularly, the glycolytic enzymes hexokinase (HK), phosphofructokinase (PFK), lactate dehydrogenase (LDH) (Cota-Ruiz et al., 2015; Ulaje et al., 2019), and glyceraldehyde-3-phosphate dehydrogenase (GAPDH) (Camacho-Jim´enez et al., 2018) can be induced differentially in tissues of *L. vannamei* in response to hypoxia.

*L. vannamei* is one of the main species in the global shrimp aquaculture business with important economic value. However, due to the rapid economic development of coastal zones, the higher frequency of hypoxia caused an increase in expenses for rearing diets. Hypoxia has become one of the major problems that affect the normal growth and development of *L. vannamei*, and it seriously restricts the sustainable aquaculture of the species.

Additionally, the muscle tissue of *L. vannamei* takes up most of the body mass and uses a lot of oxygen, however, the responses of shrimp muscle tissues to hypoxia remain unknown. Therefore, deep investigation of the hypoxia stress response of muscle tissue, THC, HC and related genes, which can not only enrich a general understanding of the hypoxia effect and mechanisms of hypoxia tolerance, but also can provide novel insights into the assisting breeding of new shrimp strains with higher resistance to low oxygen conditions.

## Materials and methods

### Shrimp and hypoxia stress

The shrimp (13 ± 0.5 cm) used in the experiment were all from Hainan Guangtai Marine Culture Co., Ltd. (Wenchang City, Hainan Province, China). Only shrimp in the inter-molt stage and similar size of juvenile shrimp were used in the experiments. Before the formal experiment, they were acclimated in the aquarium for 3 days, during which the seawater salinity was 1.9% ± 0.2%, the pH value was 8.1 ± 0.2, and the temperature was 27°C ± 1°C. Food was given twice a day (no feeding during molting and hypoxic stress), and half of the seawater was changed in the bucket every day. The content of DO under hypoxic stress was 0.5 ppm, and the stress times were 0 p.m., 3 p.m., 6 h, and 12 h. The level of dissolved oxygen was maintained by filling the barrel with nitrogen.

Two different strains of *L. vannamei*, namely Zhengda and A6410 were selected for this study. The former is hypoxia-sensitive strain and the latter is hypoxia-tolerant strain. Study has shown that the HIF-1 (Hypoxia-inducible Factor 1) expression quantity of the strain Zhengda was always higher than strain A6410 in the whole phase of hypoxia, the A6410 strain did not need more HIF-1 expression to regulate target genes to deal with hypoxic stress compared to strain Zhengda at the same level of hypoxia, which indicated that strain A6410 has better hypoxia tolerance than strain Zhenda (Wei L et al., 2016).

### Hemolymph collection and measuring of total hemocyte counts and hemocyanin concentrations

Three shrimps were randomly selected from each replicate, and there have three replicates at different hypoxia treatments, which used to measure the parameter of THC and HC. Pericardial blood was drawn from shrimp with a 1 ml syringe and mixed with anticoagulants (30 mM trisodium citrate, 0.34 M sodium chloride, 10 mM EDTA, and 0.115 M glucose) of the same volume on the ice. A hemolymph volume of 10 µl was absorbed by a microtransfer gun and counted under a light microscope, and the appropriate amount of hemolymph was centrifuged at 4°C and 5,000×g for 10 min. Then, 100 µl supernatant was taken and mixed with 2,900 µl of buffer solution (50 mM Tris, 10 mM CaCl_2_, and pH = 8.0). The absorbance values of the diluted plasma were measured at 335 nm using a UV spectrophotometer (1 cm path length) (PerkinElmer Lambda 25). The hemocyanin concentration (unit: mg.ml^−1^) was calculated using the following formula: E_335__nm_ (mg.ml^−1^) = 2.3×OD_335 nm_ (E stands for HC; 2.3 is the extinction coefficient of hemocyanin for mg.mL^−1^) (Yang and Pan 2013; Wei Y et al., 2016).

### Protein extraction and digestion

The muscle of the three individuals from each treatment were mixed equally, so total nine samples for each group were subjected to protein extraction. The muscle tissues of two strains of *L. vannamei* after 0 h and 12 h hypoxia were selected respectively as proteomics experimental materials. For the convenience of bioinformatics analysis of data, the samples of hypoxia with 0 h were defined as control I and experiment I respectively. Correspondingly, samples of hypoxia with 12 h were defined as control II and experiment II respectively. The sample was ground with liquid nitrogen, transferred to precooled cracking buffer (8 M urea, 40 mM Tris-HCl or TEAB with 1 mM PMSF, 2 mM EDTA, and 10 mM DTT, pH = 8.5), and ultrasonically treated for 2 min to release proteins. After centrifugation at 25,000×g for 20 min at 4°C, the supernatant was transferred to a new test tube, reduced with 10 mM dithiothreitol (DTT) at 56°C for 1 h, and alkylated for 45 min in the dark with 55 mM iodoacetamide (IAM) at room temperature. After centrifugation (25,000×g, 4 °C, 20 min), the protein-rich supernatant was quantified by a standard Bradford protein assay. The extracted protein samples were analyzed by SDS–PAGE electrophoresis with Coomassie brilliant blue gel staining. The protein solution (100 µg) with 8 M urea was diluted 4-fold with 100 mM TEAB. Trypsin Gold (Promega, Madison, WI, United States) was used to digest the proteins at a protein: trypsin ratio of 40:1 at 37°C overnight. After trypsin digestion, peptides were desalted with a Strata X C 18 column (Phenomenex) and vacuum-dried according to the manufacturer’s protocol.

### Labeling and grading of polypeptides

After trypsin digestion, the peptides were dissolved by adding 30 µl of 0.5 M TEAB, and the iTRAQ labeling reagents were transferred and combined with samples at room temperature. Peptide labeling was performed using the iTRAQ reagent 8-Plex kit, according to the manufacturer’s operating procedures. Labeled peptides of different reagents were desalted with a combination of Strata X C18 columns (Phenomenex) and vacuum-dried according to manufacturer specifications. The peptides were separated by the Shimadzu LC-20AB HPLC Pump system coupled with a high pH RP column. The peptides were reconstituted with buffer A [ACN:H_2_O (1:19), pH = 9.8 adjusted with ammonia] to a total volume of 2 ml and loaded onto a column containing 5 μm particles (Phenomenex). The peptides were separated at a flow rate of 1 ml/min in the following sequence: 5% buffer B [H_2_O:ACN (1:19), pH = 9.8 adjusted with ammonia] for 10 min, 5%–35% buffer B for 40 min, and 35%–95% buffer B for 1 min. The system was maintained in 95% buffer B for 3 min and then in 5% buffer B for 1 min before equilibration with 5% buffer B for 10 min. Elution was monitored by measuring the absorbance at 214 nm, and its fractions were collected every minute. The eluted peptides were pooled as 20 fractions and dried by vacuum.

### HPLC analysis

Each fraction was resuspended in buffer A (2% ACN and 0.1% FA in water) and centrifuged at 20,000×g for 10 min. The supernatant was loaded onto a C18 trap column at 5 μl/min for 8 min using an LC-20AD nano-HPLC instrument (Shimadzu, Kyoto, Japan) by an autosampler. The peptides were eluted with a trap column and then separated by an analytical C18 column (inner diameter 75 μm) packed in-house. The gradient was run at a rate of 300 nl/min starting with 8%–35% buffer B (2% H_2_O and 0.1% FA in ACN) for 35 min and then 60% buffer B for 5 min followed by 80% buffer B for 5 min. At the final stage, 5% buffer B was used for 0.1 min and equilibrated for 10 min.

### Bioinformatics analysis

High-resolution mass spectrometry data were used for further analysis. The DDA data were evaluated using MaxQuant’s integrated Andromeda engine with further spectrum library generation with Spectronaut. For large-scale DIA data, Spectronaut was used constructed spectral database information to complete deconvolution extraction of data, and the mProphet algorithm was used to complete quality control of data analysis by obtaining a large number of reliable quantitative results. GO, COG, and pathway annotation analysis were also performed during this step. The cohort of differentially expressed proteins among different comparison groups was identified based on these results.

### Total RNA extraction, reverse transcription, and quantitative real-time-PCR

The muscle of the three individuals from each treatment were mixed equally, so total nine samples for each group were subjected to total RNA extraction. The muscle tissues of two strains of *L. vannamei* after 0 h and 12 h hypoxia were selected respectively as the experimental materials used in RNA extraction, which were consistent with those used in proteome analysis. RNAiso Plus (TaKaRa) was used for total RNA extraction following the manufacturer’s protocol. The obtained RNA samples were treated with DNase I (Promega) to remove contaminating DNA. Next, approximately 2000 μg of total RNA was reverse transcribed into cDNA using a GoScript reverse transcription system (Promega) in a 25 μL reaction mixture. The expression of the hemocyanin, chitinase, HSP90, PDCD4, and GP genes was individually determined with quantitative real-time-PCR (qRT–PCR). SYBR green Master I (Roche) was used to perform qRT–PCR using obtained cDNA samples (2 μl) in a 20 μl reaction mixture on a ROCHE LightCycler 96 Real-Time Cycler PCR Detection System (Roche Applied Science, Mannheim, Germany) using the following primers (Table 1). Ribosomal protein L8 was chosen as a reference housekeeping gene (Rojas-Hernandez et al., 2019). qRT–PCR was performed with the following cycling conditions: 94°C, 10 min; (94°C, 15 s; 60°C, 1 min) × 40 cycles. All samples were examined in triplicate on the same plate. qRT–PCR data were normalized using ribosomal protein L8 expression as a reference gene (Rojas-Hernandez et al., 2019). qRT–PCR data were analyzed using the 2^-△△Ct^ method (Livak and Schmittgen 2001) and expressed as an n-fold value against the control sample.

**TABLE 1:** Primer sequence of the selected genes.

Genes	primers (5′ to 3′)	Accession numbers
*L8*	F:TAGGCAATGTCATCCCCATT	DQ316258.1
	R:TCCTGAAGGAAGCTTTACACG	
*Hemocyanin*	F:AGTGGGCATCCTTTGTCGG	KY695246.1
	R:CTGTTGGTGAAGAGGTGCGG	
*Chitinase*	F:ATCGCAACCCATCAAACCTCG	AF315689.1
	R:ACAATCGTCGCAGACACGGT	
*HSP 90*	F:GGGTCACGTCCAACAGCAAC	QCYY01001690.1
	R:TCGCCTTCACAGACACMGAGC	
*PDCD4*	F:GATTAACTGTGCCAACCAGTCCAAAG	XM_027364270.1
	R:CATCCACCTCCTCCACATCATACAC	
*GP*	F:CCAGAATCCTCCACATAACT	MK721970.1
	R:GGAATACTGGCTCCATCAC	

## Results and discussion

### Physiological responses of two strains under the hypoxic stress

**FIGURE 1:**
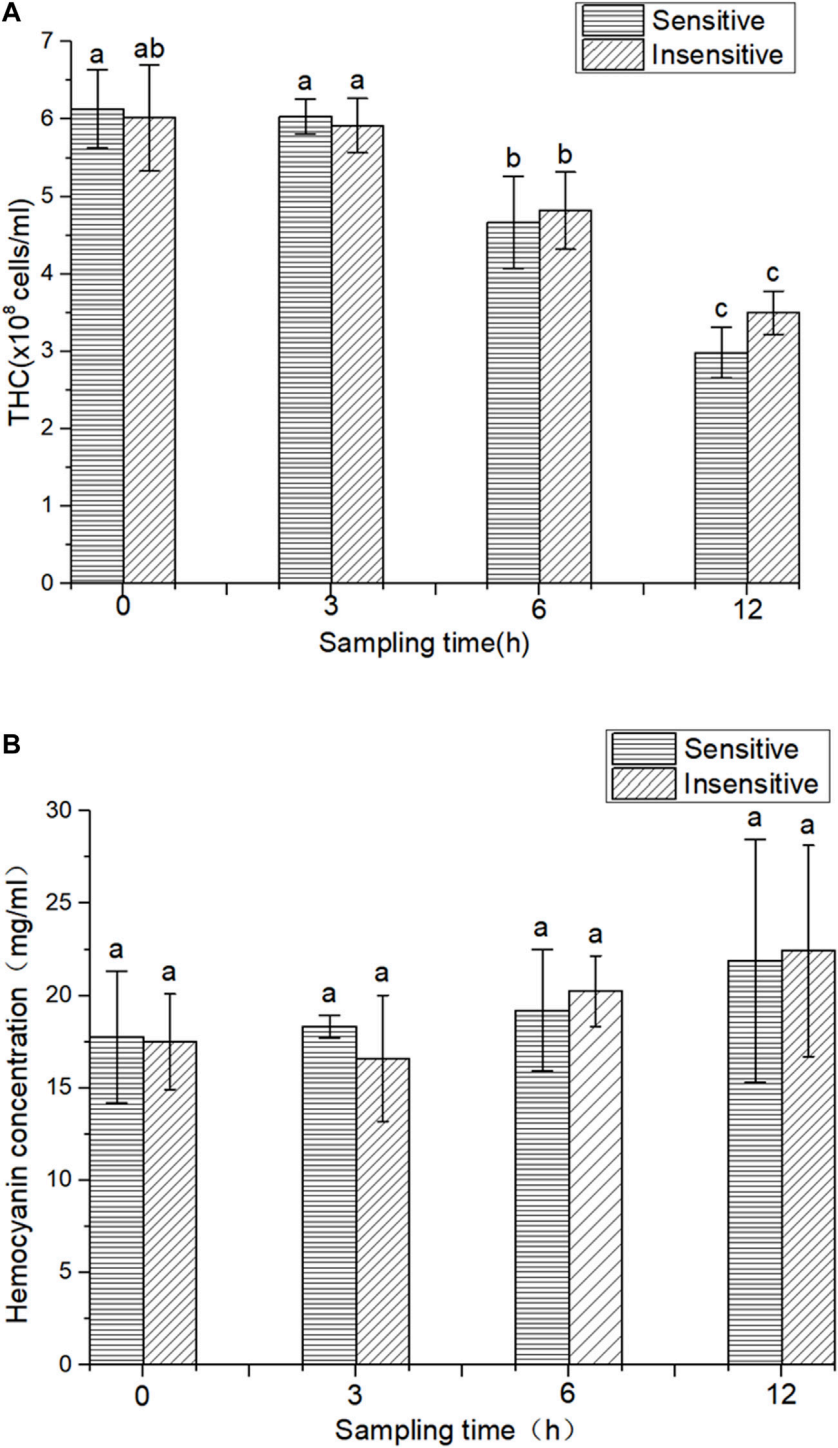
THC and HC in two strains of *L. vannamei*. **(A)** THC in two strains of *L. vannamei,* **(B)** HC in two strains of *L. vannamei*. Each bar represents the mean value of three determinations. The same letters in the data bar indicate no significant difference (*p* > 0.01), while different letters indicate significant difference (*p* < 0.01).

In this study, overall, there was no significant change in THC of the two strains after 3 h of hypoxic stress compared to 0 h. However, after 6 and 12 h of hypoxic stress, the THC parameters of the two strains were significantly reduced (*p < 0.05*). Compared with 3 h of hypoxia, 6 h of hypoxia significantly decreased the THC parameter (*p < 0.05*). Compared with hypoxia for 6 h, THC decreased significantly after 12 h of hypoxia (*p < 0.05*). The THC of the hypoxic-sensitive strain was significantly lower than that of the hypoxic-tolerant strain after 12 h and 6 h of hypoxia treatment. However, there was no significant difference in THC content between the two strains at the same time of hypoxia treatment (3, 6, and 12 h) (*p > 0.05*) (Figure 1A). The HC of hypoxic and sensitive strains showed an overall upward trend, but compared with 0 h, hypoxic treatment for 3, 6, and 12 h had no significant effect on HC (*p > 0.05*) (Figure 1B).

### Principal component analysis and sample correlation analysis

**FIGURE 2:**
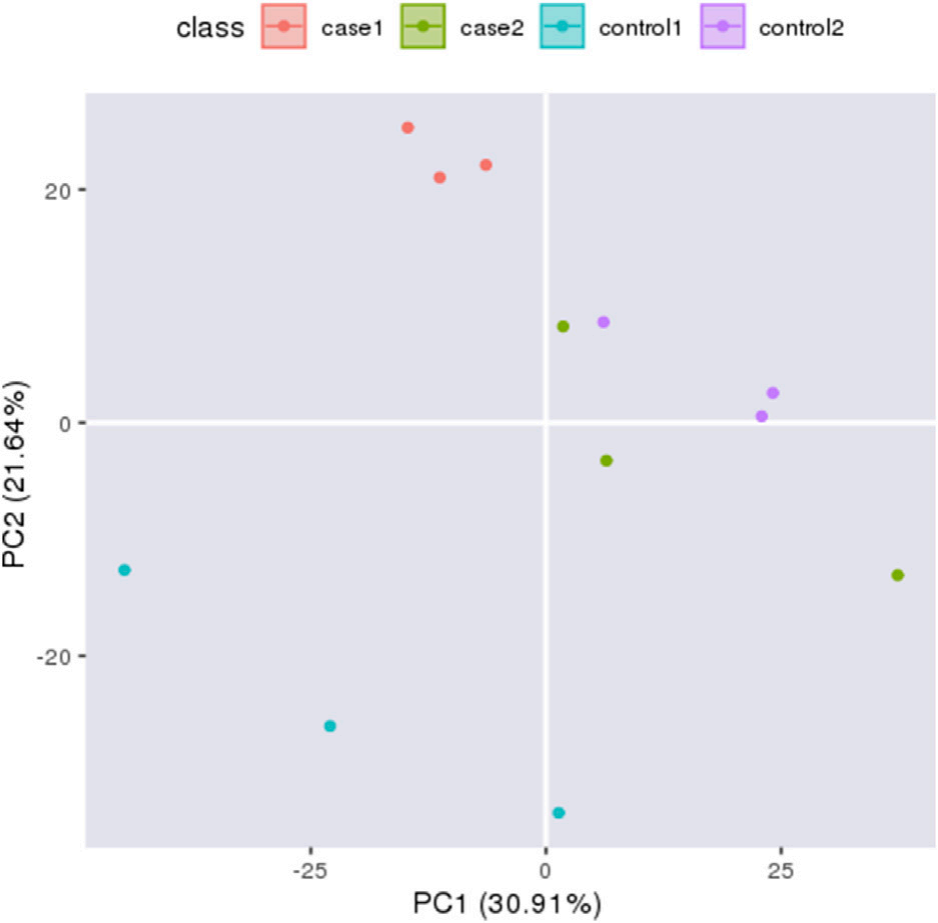
Principal Component Analysi *X*-axis displays the first principal component and *y*-axis displays the second principal component. The orange circles indicates experiment I, the green circles indicates experiment II, the blue circles indicates control I and the purple circles indicates control II.

Principal component analysis (PCA) can reflect the variability between and within groups through the original data and present the trend of intergroup separation in the experimental model. To master the aggregation and separation of experimental Group I, experimental group II, control I, and control group II experimental groups, four histone protein expression datasets were treated as four variables and analyzed by PCA with SPSS software (Figure 2). The analysis results show good independence of the four groups of variables, so four groups of data can be used for subsequent comparative analysis. To quantitatively reflect the correlation between the four groups of proteins, the Pearson correlation coefficient of protein expression between each group was calculated by SPSS software and presented in the form of a heat map (Supplementary Figure S1). Pearson correlation coefficients of protein expression in all four groups were between 0.9 and 1.0, which indicated a strong correlation between all groups.

### Statistical analysis of differential proteins

The extraction of ion peak areas was first performed by Spectronaut software, and the MSstats software package was used to calibrate and normalize the data within the system. In this study, three comparison groups were set up, namely, experimental group II vs. experiment I, control group II vs. control I, and experimental group II vs. control group II, and the differences in the expression of various comparison histones were assessed according to the set comparison group and the linear mixed effect model. When the condition of fold change ≥1.5 and corrected *p* value (adj_*p* value) < 0.05 was met, the difference was considered significant.

In this study, four groups of protein expression data were first analyzed by data-dependent acquisition (DDA) mass spectrometry and all detectable nonredundant high-quality MS/MS spectrogram information was obtained after database identification in MaxQuant software, which was used as the spectrogram database for subsequent DIA (Supplementary Figure S2). The total number of peptide and the number of protein detected in the three comparison groups were 16,603 and 3417, respectively. A total of 1,452 proteins were detected in the experimental group II vs experimental group I comparison group; among them, 1,417 proteins had no significant difference in expression level (*p > 0.05*), 10 proteins were significantly upregulated, and 25 proteins had downregulated expression levels (*p < 0.05*). In the control group II vs control group I comparison group, a total of 1,448 proteins were detected, among which 1,175 proteins had no significant difference in expression level (*p > 0.05*), 29 proteins were significantly upregulated, and 244 proteins were significantly downregulated (*p < 0.05*). In contrast, among the 1,525 proteins detected in the experimental group II vs control group II comparison group, 1,460 detected proteins had no significant difference in expression (*p > 0.05*), 49 proteins had significantly upregulated expression, while 16 proteins had significantly downregulated expression (*p < 0.05*). The volcanogram illustrates the differential protein expression in the three comparison groups in a more intuitive manner (Supplementary Figure S3).

### Gene ontology classification of differential proteins

**FIGURE 3:**
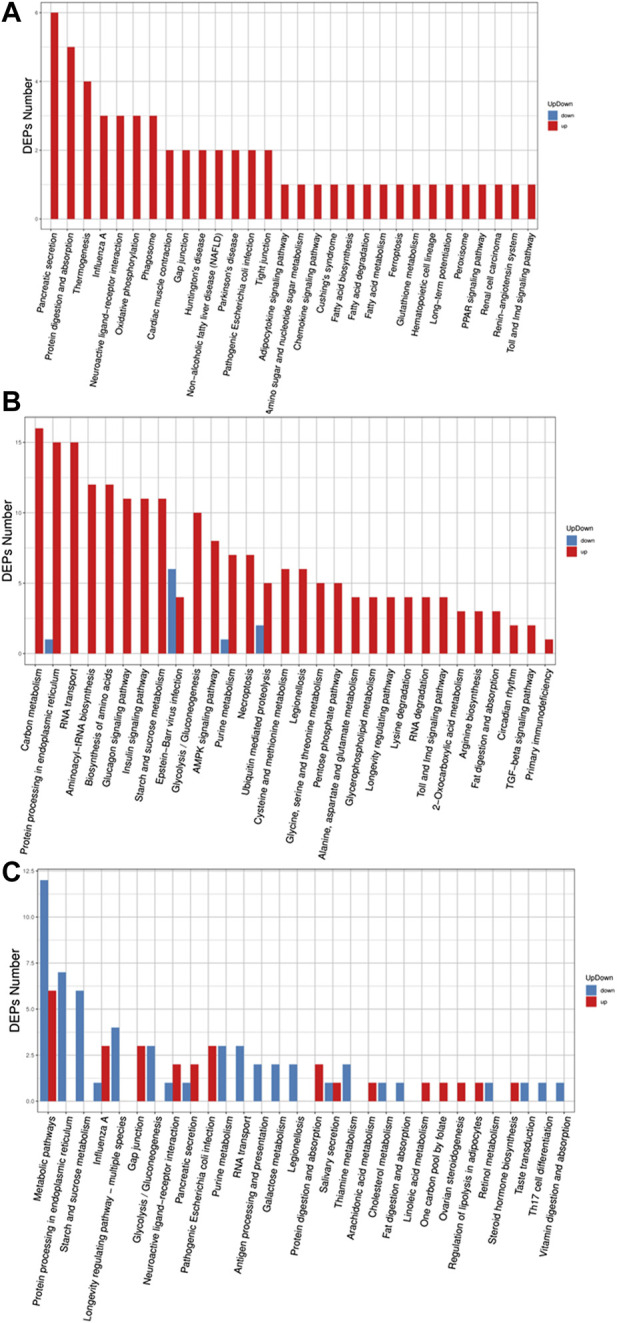
Barplot of the Gene Ontology analysis. **(A)** experiment II vs. experiment I, **(B)** control II vs. control I, **(C)** experiment II vs. control II. The bar chart shows the distribution of corresponding GO terms. Different colors represent different GO categories.

In the GO (Gene Ontology) classification diagram of experimental group II vs. experimental group I, proteins in experimental group II related to biological process, cellular component, and molecular function category, were mostly upregulated compared to experimental group I, such as genes involved in signaling, metabolic process, response to stimuli, regulation of the biological processes, and membrane systems (Figure 3A). However, all differentially expressed proteins in the category of multicellular biological processes were downregulated. In the GO classification diagram of control group II vs. group I, control group II had upregulated proteins mostly from the category belonging to biological process, cellular component, and molecular function. (Figure 3B). Among them, proteins were identified that were involved in responses to stimuli, negative and positive regulation of the biological processes, signal transduction, growth, immune system processes, locomotion, pigmentation, membrane-enclosed lumen, extracellular regions, supramolecular complex, cell junction, and antioxidant activity. In the GO classification diagram of experimental group II vs control group II, some proteins were related to biological processes, cellular components, and molecular functions, while the other part was downregulated in control group II compared with experimental group II (Figure 3C). Among the upregulated proteins were those involved in cellular component organization or biogenesis, organelles, supramolecular complexes, molecular transducer activity, and structural molecule activity. However, the expression of differential proteins associated with the stress response, extracellular region part, and membrane were downregulated.

### Eukaryotic orthologous groups classification of differential proteins

**FIGURE 4:**
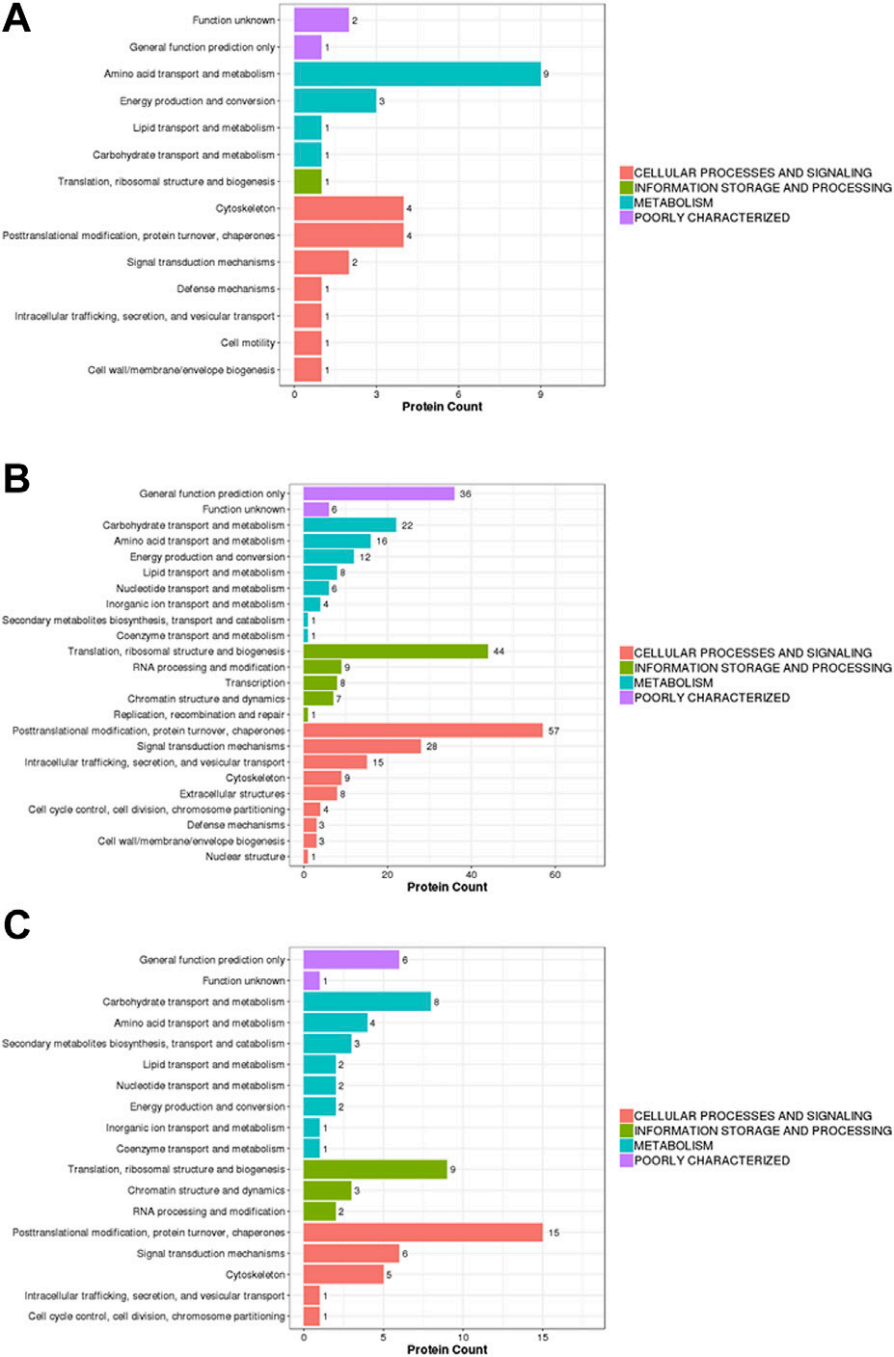
Barplot of the KOG analysis. **(A)** experiment II vs. experiment I, **(B)** control II vs. control I, **(C)** experiment II vs. control II. Eukaryotic orthologous groups (KOGs) were delineated by comparing protein sequences encoded in complete genomes, representing major phylogenetic lineages. *X*-axis displays the KOG term, *y*-axis displays the corresponding protein count illustrating the protein number of different function.

In this study, the identified proteins were compared with the KOG (eukaryotic orthologous groups) database to predict and classify their possible functions. In the KOG classification diagram of experimental group II vs. experimental group I, the main difference among the proteins was associated with post-translational modification function (amino acid transport and metabolism) and the cytoskeleton, as well as post-translational modification and protein turnover (chaperones) (Figure 4A). In the KOG classification diagram of control group II vs. control group I, in addition to proteins with uncertain functions, there were many differences within proteins involved in post-translational modification, protein turnover, chaperones, translation, ribosome structure and biogenesis, and signal transduction mechanisms (Figure 4B). In the KOG classification diagram of experimental group II vs. the control group, most proteins were related to post-translational modification, protein turnover, chaperones, translation, ribosomal structure and biogenesis, carbohydrate transport, and metabolism (Figure 4C).

### Expression analysis of important functional protein genes under the hypoxic stress in *L. Vannamei*

**FIGURE 5:**
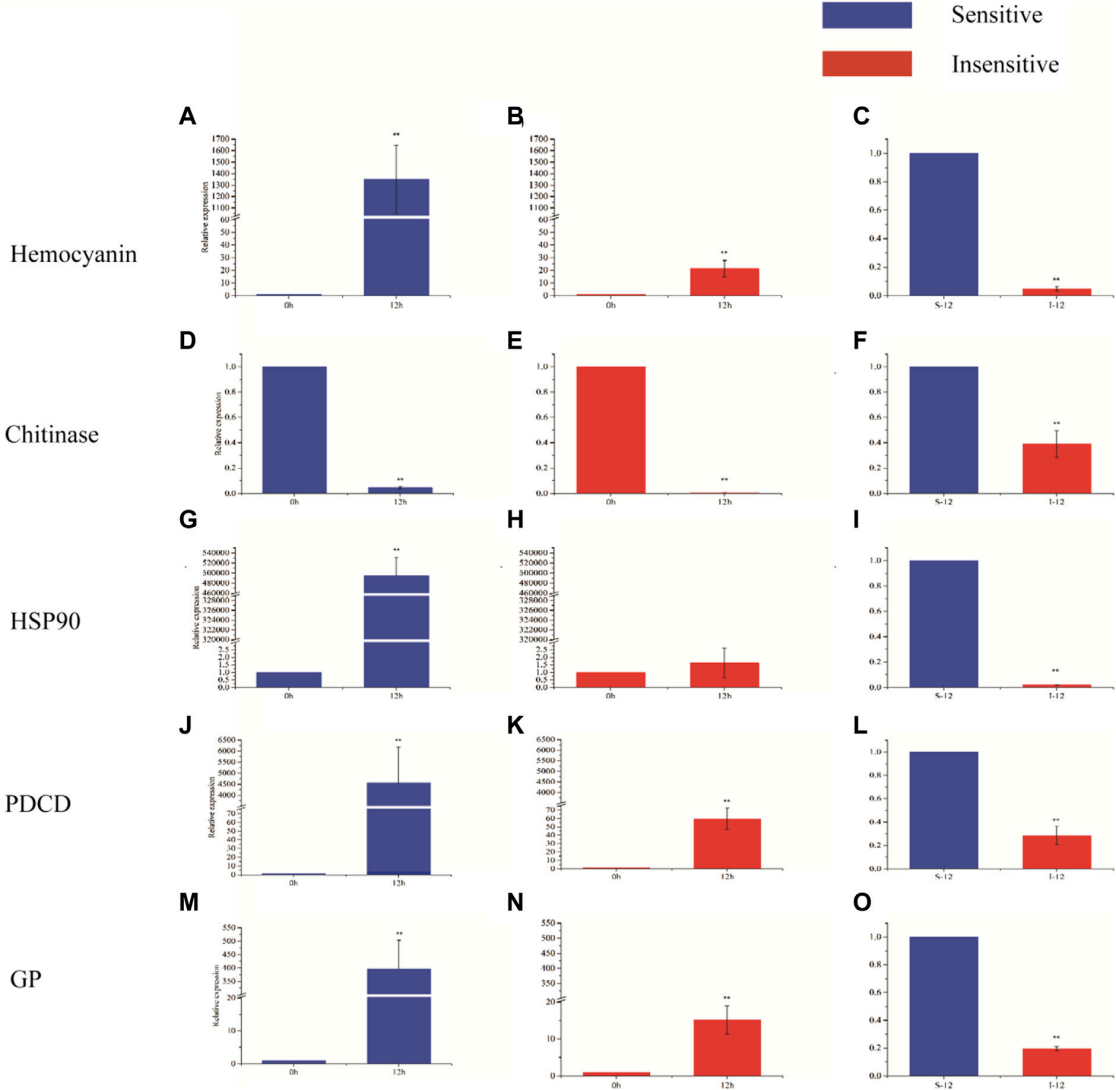
Expression of five genes in different periods of hypoxia in two strains of *L. vannamei*. *X*-axis displays the KOG term, *y*-axis displays the corresponding protein count illustrating the protein number of different function.

Hemocyanin gene, chitinase gene, heat shock protein 90 gene, programmed cell death protein gene and glycogen phosphorylase gene were selected for expression analysis (Figure 5). After 12 h of hypoxia stress, the expression levels of hemocyanin gene, programmed cell death protein gene and glycogen phosphorylase gene were significantly increased in hypoxia-sensitive and hypoxia-tolerant families (*p* < 0.05), and the expression levels of these genes were significantly different in the two families (*p* < 0.05). The expression of chitinase gene in the two families was significantly decreased (*p* < 0.05), and the expression of chitinase gene was significantly different (*p* < 0.05). The expression level of heat shock protein 90 gene in hypoxic-sensitive family was significantly increased (*p* < 0.05), while the expression level of heat shock protein 90 gene in hypoxic-resistant family was not significantly changed (*p* > 0.05).

## Discussion

The dynamic changes in protein expression in the muscle tissue of *L. vannamei* under hypoxic stress were studied by the iTRAQ technique. A total of 3417 proteins were detected. The possible functions of all identified proteins were annotated according to GO, KEGG, and DEPS databases. By comparing the proteome of the control group and the experimental group, detailed information about the proteome response to hypoxic stress could be obtained. Low oxygen levels affect the immune function of *L. vannamei*. Crustaceans have nonspecific immunity, where hemocytes are the main effector of the immune response (Huang and Ren, 2019). Hemocytes have the ability to wrap, engulf, and degrade invading pathogens and play a crucial role in crustacean immune defense (Liu et al., 2020). A number of animal hemocytes often change in response to environmental changes or pathogenic microorganism infection (Qiu et al., 2011), so they are a marker of body health and immune capacity. Under the condition of low oxygen (1.5 PPM), THC of green-lipped mussel (*Perna viridis*) (Wang et al., 2012) and scallop (*Chlamys farreri*) (Chen et al., 2007) decreased gradually with decreasing DO value. In this experiment, the THC of the two strains of *L. vannamei* showed a downward trend at 0 h and 3 h after hypoxia treatment, without reaching a significant difference. THC was significantly decreased at 6 and 12 h after hypoxia treatment (*p* < 0.05), which was similar to the above results.

Hemocyanin is the most important plasma protein of crustaceans and can bind and transport O_2_ and CO_2_ to serve as the respiratory protein of prawns. In addition to its main function as an oxygen carrier, hemocyanin has been identified as a nonspecific innate immune defense molecule of crustaceans (Coates and Nairn 2014) with antiviral and antibacterial properties (Lee, Lee and Soderhall 2003). It can be functionally converted into phenolic oxidase with agglutination abilities and hemolytic activity (Zhang et al., 2006). When oxygen levels in the environment are low, crustaceans meet their oxygen needs by increasing the concentration of hemocyanin. A previous study revealed that HC in *L. vannamei* was significantly increased (*p < 0.05*) under hypoxic conditions (Wei L et al., 2016). Another shrimp species, oriental river prawn (*Macrobrachium nipponense*), had significantly increased (*p < 0.05*) expression levels of hemocyanin in response to the hypoxic environment (Sun et al., 2016). In contrast, 10 h of hypoxic conditions negatively affected the HC rate in the southern king crab (*Lithodes santolla*) (Paschke et al., 2016).

In this study, hypoxia treatment had no significant effect on the HC of *L. vannamei* in the two strains with hypoxia tolerance and sensitivity (*p > 0.05*), and there was no significant difference in HC between the two strains at different periods of hypoxia (*p > 0.05*). However, HC showed an overall upward trend compared to the control group, consistent with previous reports. In addition, HC increased gradually with prolonged hypoxia time, which was consistent with proteomic data.

Under low DO circumstances, shrimp can adjust the use of energy substrates (carbohydrate, lipids, and proteins) to balance oxidative (Ulaje et al., 2019). In this experiment, immune-related proteins, such as hemocyanin, chitinase, and heat shock protein 90, were found among the proteins expressed at significantly different levels under hypoxic stress. Hemocyanin plays an important role in the innate immunity of *L. vannamei*, such as antibacterial, antiviral, hemolytic, anti-infective, and antitumor activities (Jiang et al., 2007; Zhang et al., 2009; Coates and Nairn 2014; Zheng et al., 2016). Chitinases are widely exist in organisms as a group of hydrolytic enzymes that hydrolyze chitin. The function of chitinases in biological processes such as the growth of fungi, the molting of arthropods, and the invasion of bacteria or parasites into chitincontaining structures of the host has been intensely studied (Arakane and Muthukrishnan, 2010; Chaudhuri et al., 2010; Pesch et al., 2016). Chitinase is a key enzyme in the innate immunity of *L. vannamei* and involved in numerous immunomodulatory responses (Zhang et al., 2016; Niu et al., 2018; Song et al., 2020), especially in preventing bacterial infection (Duo-Chuan 2006; Gao et al., 2017). Chitinase expression in *L. vannaensis* infected with white spot syndrome virus is upregulated at the translation level (Jiang et al., 2007). A previous study demonstrated that chitinase plays a role in regulation of both humoral and cellular immune responses in shrimp due to the expression of various immune related genes and other functional proteins with antibacterial and antiviral activities was widely changed in LvChi5 silencing shrimp (Niu et al., 2018).

Heat shock proteins are an important molecular chaperone in eukaryotic cells (Zininga, Ramatsui and Shonhai, 2018). They play a role in protecting cells from stress and oncogenic transformation, providing cell cycle regulation, antigen presentation, and participation in cellular stress responses, including changes in environmental conditioning stress (Kühl and Rensing 2000; Udono 2012; Wu et al., 2017; Sornchuer et al., 2018). It helps to refold the denatured protein into an appropriate conformation (Nakamoto et al., 2014).

In this study, Hsp90 was significantly upregulated in hypoxic-sensitive *L. vannamensis* after 12 h of hypoxia stress, while there was no significant change in the expression of Hsp90 in hypoxic-tolerant families. HSPs have been shown to be one of the main response proteins to hypoxic stress (Zhang, Zhang and Zhang 2016; Niu et al., 2018). Although Ulaje *et al.* showed that Hsp70 and Hsp90 gene expression in *L. vannamei* was down-regulated under hypoxia, in both the short- and the long-term (Ulaje et al.,2020), most researches in crustaceans have indicated that the up-regulation in the expression of Hsps genes is a general response to cope with hypoxia (Sun et al., 2014, 2016; Jolly et al., 2018), which was consistent with the results of this study.

The hypoxic-sensitive strain *L. vannamensis* can regulate the protein level in a timely manner in response to the hypoxic-sensitive strain, while the protein expression in the hypoxic-tolerant strain is at a normal level. In addition, after 12 h of hypoxia, the expression of neuroendocrine differentiation factor in the hypoxic sensitive family was significantly upregulated, which may be related to its role in immune regulation (Sung et al., 2016; Song et al., 2020; Junprung et al., 2017). The role of other proteins identified in this study as a part of the response to hypoxia stress in *L. vannamei* remains to be further studied.

## Conclusion

Hypoxia stress has become a frequent occurrence in commercial *L. vannamei* farming, so it is important to explore the molecular mechanisms of the hypoxic response and adjustment to changing oxygen levels. This study demonstrated the changes in physiological and biochemical levels in shrimp under conditions of low oxygen stress and investigated the expression of the hypoxic stress protein regulation mechanism and its function by comparing proteomics data among two strains of *L. vannamei* with different tolerances to hypoxia. The results from proteomic analysis were confirmed with qRT–PCR to detect the gene expression level.

Studies have indicated that low oxygen levels have an effect on THC and HC parameters. The hypoxia-sensitive strain showed a decreased number of hemocytes after 3 h under hypoxic conditions, while the hypoxia-tolerant strain response with significant changes in hemocyte number was delayed to 6 h in a hypoxic environment. Since hemocytes are involved in oxygen transportation together with the immune response, these results suggest the weakening of immune system capacity in response to low oxygen levels.

A total of 3417 proteins were detected in proteomics analysis. The hypoxia-sensitive strain showed 273 differentially expressed proteins in response to 12 h hypoxia treatment, while in the hypoxia-tolerant strain, this number was reduced to 35 proteins. The cohort of proteins that were affected in the two strains included hemocyanin, Hsp90, GP, chitinase, PD, actin, ferritin, and trypsin. These proteins were classified into immune-related proteins, energy metabolism-related proteins, cytoskeleton-related proteins, chaperones, and others.

Five protein genes with significant changes at the proteomic level in two strains of *L. vannamei* were chosen for qRT–PCR to confirm the gene expression patterns, namely, hemocyanin, chitinase, HSP90, PD, and GP. This group of proteins is probably an important component of the *L. vannamei* response to hypoxia stress and could be considered biomarkers.

## Data Availability

The raw data supporting the conclusion of this article will be made available by the authors, without undue reservation.

## Supplementary material

SUPPLEMENTARY FIGURE S1 Sample correlation analysis heat map. The sign of correlation coefficient represents positive or negative correlation. The value represents the strength of correlation: 0.8–1.0 represents a strong correlation, 0.6–0.8 represents a strong correlation, 0.2–0.4 represents a weak correlation, 0.0–0.2 represents a very weak correlation or no correlation.

SUPPLEMENTARY FIGURE S2 Protein mass distribution. *X*-axis displays the protein mass interval (Kilodalton), *y*-axis displays the corresponding protein number.

SUPPLEMENTARY FIGURE S3 Differential protein volcano map. experiment II-vs. -Experiment I, **(B)** control II-vs.-control I, **(C)** experiment II-vs.-control II. X-axis of the volcanogram refers to the multiple protein fold change difference (log2), and the Y-axis corresponds to -log10 (P value). The green circle indicates the proteins with significantly downregulated patterns, the red circle indicates the proteins with significantly upregulated patterns, and the gray circle indicates the proteins with no significant difference.
