# Supplementary material for: Physiological and muscle tissue responses in Litopenaeus vannamei under hypoxic stress via iTRAQ
Source: Front Physiol. 2022 Aug 30;13:979472. doi: 10.3389/fphys.2022.979472 (PMC9468788; doi:10.3389/fphys.2022.979472)
Supplement: Supplementary file 1 [file Table1.DOCX]

| genes | primers（5' to 3'） | Accession numbers |
| --- | --- | --- |
| *L8* | F：TAGGCAATGTCATCCCCATT | DQ316258.1 |
|  | R：TCCTGAAGGAAGCTTTACACG |  |
| *Hemocyanin* | F：AGTGGGCATCCTTTGTCGG | KY695246.1 |
|  | R：CTGTTGGTGAAGAGGTGCGG |  |
| *Chitinase* | F：ATCGCAACCCATCAAACCTCG | AF315689.1 |
|  | R：ACAATCGTCGCAGACACGGT |  |
| *HSP 90* | F：GGGTCACGTCCAACAGCAAC | QCYY01001690.1 |
|  | R：TCGCCTTCACAGACACMGAGC |  |
| *PDCD4* | F：GATTAACTGTGCCAACCAGTCCAAAG | XM_027364270.1 |
|  | R：CATCCACCTCCTCCACATCATACAC |  |
| *GP* | F：CCAGAATCCTCCACATAACT | MK721970.1 |
|  | R：GGAATACTGGCTCCATCAC |  |
