# Supplementary figures and images for: Physiological and muscle tissue responses in Litopenaeus vannamei under hypoxic stress via iTRAQ
Source: Front Physiol. 2022 Aug 30;13:979472. doi: 10.3389/fphys.2022.979472 (PMC9468788; doi:10.3389/fphys.2022.979472)

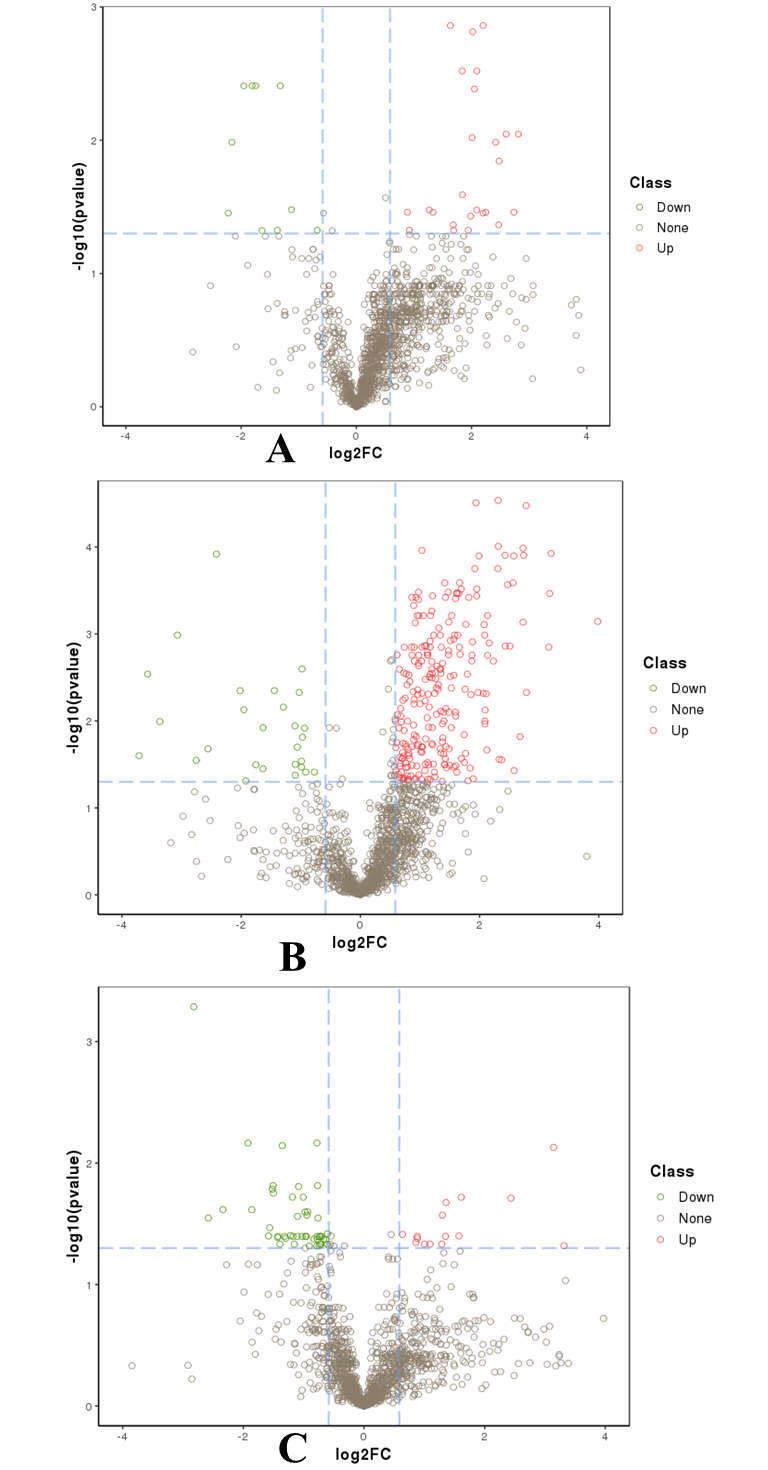

Supplement: Supplementary file 2 [file Image3.TIF]

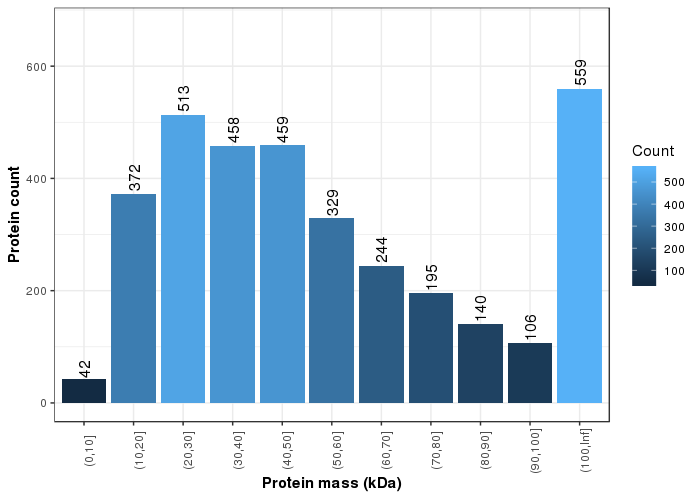

Supplement: Supplementary file 3 [file Image2.PNG]

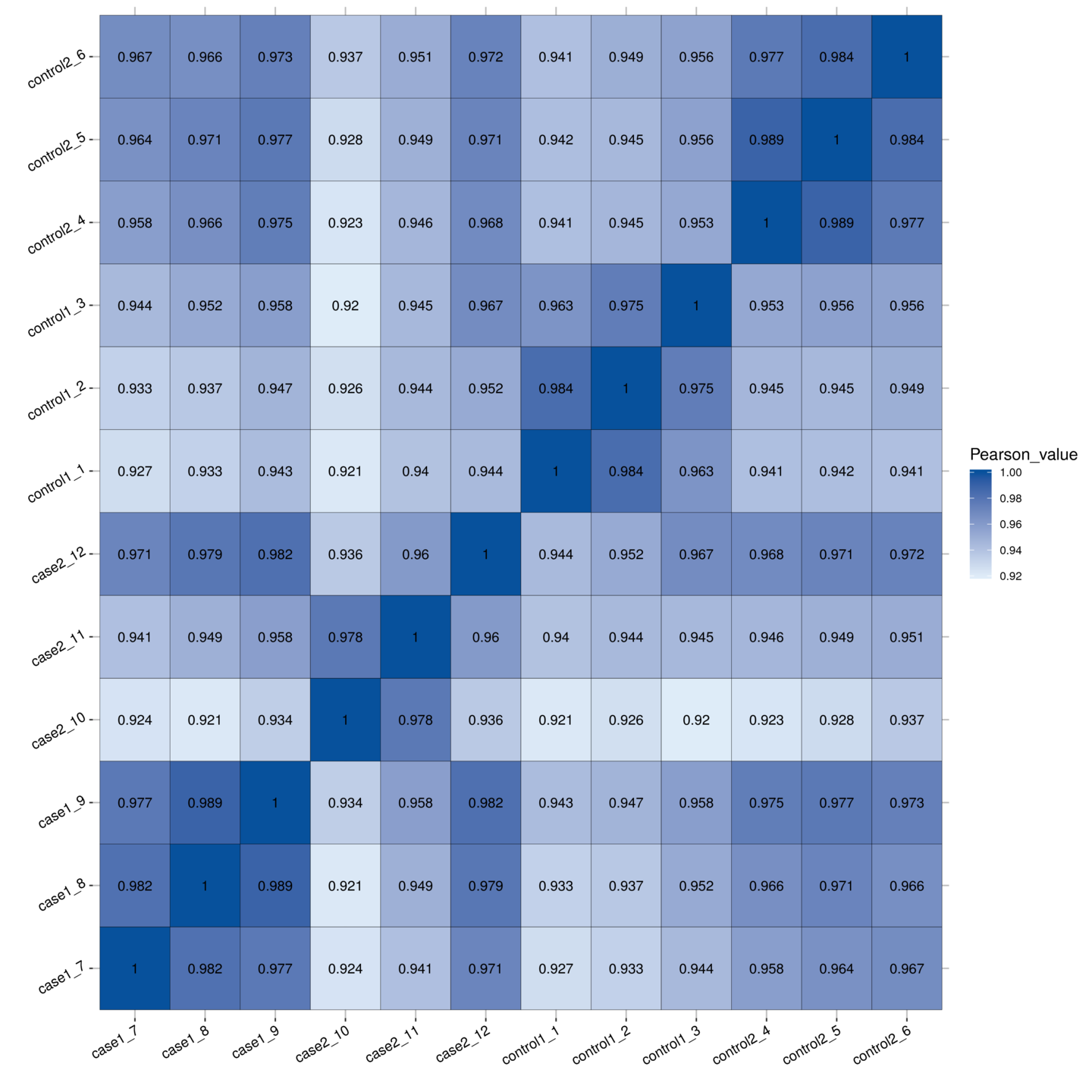

Supplement: Supplementary file 4 [file Image1.PNG]
